# Neighbourhood socioeconomic inequalities in incidence of acute myocardial infarction: a cohort study quantifying age- and gender-specific differences in relative and absolute terms

**DOI:** 10.1186/1471-2458-12-617

**Published:** 2012-08-07

**Authors:** Carla Koopman, Aloysia AM van Oeffelen, Michiel L Bots, Peter M Engelfriet, WM Monique Verschuren, Lenie van Rossem, Ineke van Dis, Simon Capewell, Ilonca Vaartjes

**Affiliations:** 1 Julius Center for Health Sciences and Primary Care, University Medical Center Utrecht (STR 6.131), P.O. Box 85500, 3508 GA, Utrecht, The Netherlands; 2 National Institute for Public Health and the Environment, Bilthoven, the Netherlands; 3 Dutch Heart Foundation, The Hague, the Netherlands; 4 Department of Public Health, University of Liverpool, Liverpool, UK

**Keywords:** Coronary heart disease, Acute myocardial infarction, Incidence, Socioeconomic status, Relative, Absolute, The Netherlands

## Abstract

**Background:**

Socioeconomic status has a profound effect on the risk of having a first acute myocardial infarction (AMI). Information on socioeconomic inequalities in AMI incidence across age- gender-groups is lacking. Our objective was to examine socioeconomic inequalities in the incidence of AMI considering both relative and absolute measures of risk differences, with a particular focus on age and gender.

**Methods:**

We identified all patients with a first AMI from 1997 to 2007 through linked hospital discharge and death records covering the Dutch population. Relative risks (RR) of AMI incidence were estimated by mean equivalent household income at neighbourhood-level for strata of age and gender using Poisson regression models. Socioeconomic inequalities were also shown within the stratified age-gender groups by calculating the total number of events attributable to socioeconomic disadvantage.

**Results:**

Between 1997 and 2007, 317,564 people had a first AMI. When comparing the most deprived socioeconomic quintile with the most affluent quintile, the overall RR for AMI was 1.34 (95 % confidence interval (CI): 1.32 – 1.36) in men and 1.44 (95 % CI: 1.42 – 1.47) in women. The socioeconomic gradient decreased with age. Relative socioeconomic inequalities were most apparent in men under 35 years and in women under 65 years. The largest number of events attributable to socioeconomic inequalities was found in men aged 45–74 years and in women aged 65–84 years. The total proportion of AMIs that was attributable to socioeconomic inequalities in the Dutch population of 1997 to 2007 was 14 % in men and 18 % in women.

**Conclusions:**

Neighbourhood socioeconomic inequalities were observed in AMI incidence in the Netherlands, but the magnitude across age-gender groups depended on whether inequality was expressed in relative or absolute terms. Relative socioeconomic inequalities were high in young persons and women, where the absolute burden of AMI was low. Absolute socioeconomic inequalities in AMI were highest in the age-gender groups of middle-aged men and elderly women, where the number of cases was largest.

## Background

Coronary heart disease (CHD) is one of the leading causes of disability and death in both high-income and low-income countries [1,2]. Both individual socioeconomic status (SES) and the socioeconomic status of the neighbourhood of residence are independently and significantly associated with incidence of acute myocardial infarction (AMI) [3,4]. The association between neighbourhood SES and AMI is generally well documented in western countries, indicating that those living in deprived areas experience the largest burden of the disease with higher incidence, [4-13] prevalence [14] and mortality [7,15] rates. Neighbourhood-level SES is often used in population-based studies where individual-level SES is not available. However, the interest in neighbourhood SES has also arisen because of the recognition of the importance of the environment in which people live for the risk of CHD.

The magnitude of socioeconomic inequalities in AMI incidence varies between countries and periods, and is related to socio-demographic factors such as age and gender [2,11,16]. In general, age and gender are the most important factors that affect health. Some studies have suggested that living in a deprived neighbourhood may affect coronary health of women to a greater extent than men [6,11-13]. Furthermore, a decrease in the socioeconomic gradient with age has been described [6,11]. However, most of the studies on the association between SES and AMI were too small or limited in age range to allow for explorations of the varying magnitude of socioeconomic inequalities across a wide range of age- and gender-groups [7,8,10,12].

Several studies have expressed socioeconomic inequalities in AMI risk in relative terms using conventional relative risk approaches. To our knowledge no study described the age pattern in socioeconomic inequalities as the absolute number of AMI events attributable to socioeconomic differences. It is important to assess whether socioeconomic inequalities affect AMI incidence in a similar way in all age-gender-groups. Relative effect measures may decrease with age, but absolute differences may not. Knowledge on the age- and gender-distribution of the disease burden of socioeconomic inequalities can provide useful insight to improve public health. The objective of this study was to examine the importance of socioeconomic inequalities in relation to first AMI in the Netherlands considering both relative and absolute measures of risk differences, with a particular focus on age and gender.

## Methods

We conducted a cohort study by linkage of national registries. Registries and linking procedures used in this study have been described in detail previously [17]. We linked data between national registers using a record identification number assigned to each resident in the Netherlands with a unique combination of birth date, gender and postal code (about 84 % of population). We identified all patients with a first AMI event by first AMI hospital admission or death between January 1997 and December 2007 for all uniquely identifiable individuals registered in the Dutch population registry. Primary discharge diagnosis in the Dutch hospital discharge register (HDR) and underlying cause of death from the cause of death register were used for this purpose. Diagnoses were coded according to the International Classification of Diseases (ICD-9 410 and ICD-10 I21, respectively). We collected information on previous hospital admissions by linkage with the HDR of 1995 and 1996. Those with previous AMI admissions were excluded. Linkage of individual data between registers was performed in accordance with the privacy legislation in the Netherlands.

SES was determined at the neighbourhood-level by mean income of the neighbourhood of residence using the regional income register (RIO). [18] The RIO is a national study that contains information on income levels of neighbourhoods based on the tax information of about one third of Dutch inhabitants. The Netherlands (*N =* 15.6 million in 1997) is subdivided into 11,412 neighbourhoods with a mean population of 1,364 inhabitants (range: 1 – 32,786). Mean equivalent household income - a measure of disposable income in proportion to the household composition - was used as SES indicator in our study. Less than 0.1 % of Dutch inhabitants had missing information on neighbourhood SES. The study population was divided into socioeconomic quintiles according to the ranking of neighbourhoods in the RIO of 1997 weighted by the number of inhabitants per neighbourhood (Q1 is the most affluent quintile and Q5 the most deprived). In this manner each quintile contained about 20 % of the person-years at risk from the Dutch population.

The number of person-years at risk from the Dutch population was calculated by age, gender and socioeconomic quintile from 1997 to 2007 and used as denominator for the incidence rate calculations. The numerators of AMI incidence cases and denominators of person-years at risk from the uniquely identifiable part of the total Dutch population were used to study socioeconomic differences in AMI incidence in relative terms. Thus, for incidence rates non-unique persons were excluded from both AMI incident cases and person-years at risk. A pilot study has suggested that non-uniqueness on the linkage variables relates to large cities, foreign origin and age, however differences are small for the determinants large cities and foreign origin [19]. To examine the impact of socioeconomic inequalities on differences in the absolute number of AMI events in the Netherlands we have inflated the number of AMI events to correct for the exclusion of non-unique persons. Adjustment for the 16 % of Dutch inhabitants who were not included in our study enabled the presentation of a nationally representative number of AMI events that was attributable to socioeconomic disadvantage. We used age-gender specific inflation factors, which were calculated on the population registry of the mid-year of the study period 2002.

### Statistical analyses

Incidence rates were calculated stratified by age, gender and socioeconomic quintile. To compare incidence rates we standardized to the European standard population. Age was stratified in 10-year age-groups with <35 years as youngest age-group and ≥95 years as oldest age-group. Absolute rate differences with 95 % confidence intervals (CI) between men and women were calculated as the difference between the two incidence rates. Relative risks (RRs) by socioeconomic quintile were obtained from Poisson regression models and presented with 95 % CI. Interaction terms between age, gender and socioeconomic quintile were added in unstratified analyses.

RRs from stratified Poisson regression in the separate age-gender groups were also used to assess the absolute magnitude of socioeconomic inequalities within the age-gender groups. We calculated population attributable risk proportions (PAR) using the extended formula for multi-category exposure, in this case the socioeconomic quintiles [20]:

(1) $$\text{PAR=}\frac{\sum_{i=0}^{k}P_{i}\left(RR_{i}-1\right)}{1+\sum_{i=0}^{k}P_{i}\left(RR_{i}-1\right)}$$

Subscript *i* refers to the *i * th exposure level (Q2, Q3, Q4 and Q5). *RR *_*i*_ is the relative risk comparing the *i * th exposure level with Q1 (*i* = 0). Factor *P*_*i*_ comprises the proportion of person-years of the specific socioeconomic quintile within an age-gender group with respect to the total number of person-years of the age-gender group. The number of excess events attributable to socioeconomic inequalities was calculated as the total number of events within the age-gender-groups (inflated for non-uniqueness) multiplied by the PAR of that age-gender group. These total excess events can be interpreted as the nationwide burden of AMI in the separate population groups that would theoretically have been *eliminated * if all persons would have had the same risk for AMI as those in the reference group Q1 (the most affluent socioeconomic quintile).

To estimate the potential impact of a population shift in AMI risk by *reducing* socioeconomic inequalities instead of eliminating, we calculated a more realistically attainable proportion of potentially preventable cases using a variant of the above formula for PAR. Here, we have chosen to use the term “preventable proportion” (PP) [21]:

(2) $$\text{PP=}\frac{\sum_{i=1}^{k}P_{i}(RR_{i}-RR_{i-1})}{1+\sum_{i=0}^{k}P_{i}\left(RR_{i}-1\right)}$$

Subsequently, the PP was multiplied by the total number of events. The outcome of this calculation can be interpreted as the reduction in number of events that theoretically would not have occurred if the population in Q5, Q4, Q3 and Q2 would have had the risk for AMI of one socioeconomic exposure-level down (i.e. less deprived). Example calculations of the PP and PAR are provided in Additional file 1. Analyses were performed using SPSS version 14.0 and *P*-values less than 0.05 were considered significant.

## Results

### Population distribution

**Figure 1:**
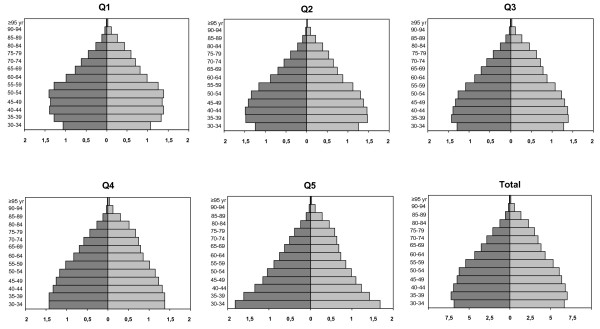
**Distribution of Dutch population from 1997 to 2007 by age, gender and socioeconomic quintile.** A total of 176,715,060 person-years is expressed in millions of person-years. Socioeconomic quintiles are ranked at the neighbourhood level according to their mean equivalent household income, quintile 1 is least deprived. Distribution of population aged <30 year is not displayed. Men (*left*), women (*right*).

The estimated total study population comprised 176,715,060 person-years of follow-up. Mean equivalent household income of neighbourhoods ranged from a median of 19,238 euro per year in the most affluent quintile, Q1, to a median of 13,096 euro per year in Q5. The populations of each socioeconomic quintile showed different distributions of age and gender, with relatively more younger persons in the most deprived quintiles (Figure 1).

### AMI incidence rates

Table 1 shows the incidence rates of AMI in the various age-gender-groups. More men than women had events (201,221 vs. 116,342), with higher event rates in every age-group. Overall, men had a 2.45-times (95 % CI: 2.43 – 2.47) higher risk for AMI than women. Age-standardized comparison of incidence rates resulted in 212 AMI events per 100,000 person-years in men, and 87 events per 100,000 person-years in women. The relative risk for AMI by gender increased up to the age of 45–54 years and subsequently decreased. The absolute rate difference between men and women continuously increased with age. In Table 2 we present age- and gender-specific incidence rates for the socioeconomic quintiles. Event rates increased with increasing socioeconomic deprivation in both men and women, in all age-groups except for the age-groups ≥95 years.

**Table 1:** Incidence of AMI per 100 000 person-years in the Netherlands between 1997 and 2007 stratified by age and gender

	**Age-group (years)**								**Age standardized rate**‡
	**<35**	**35-44**	**45-54**	**55-64**	**65-74**	**75-84**	**85-94**	**≥95**	
**Male**									
Number of events	1534	10 783	32 120	46 694	52 782	43 502	13 092	714	201 221
Incidence rate *	3.9	76	252	469	819	1272	1714	1952	212
**Female**									
Number of events	561	3032	7863	13 338	25 833	39 597	23 911	2208	116 342
Incidence rate *	1.5	22	63	136	354	737	1199	1368	87
**Male vs. Female**									
Absolute rate difference	2.4	54	189	333	465	535	515	584	126
(95 % CI)	(2.1 – 2.7)	(52 – 56)	(185 – 192)	(328 – 339)	(457 – 474)	(520 – 549)	(482 – 549)	(430 – 739)	(124 – 127)
Relative Risk **†**	2.64	3.46	4.00	3.46	2.31	1.73	1.43	1.43	2.45
(95 % CI)	(2.37 – 2.94)	(3.31 – 3.62)	(3.89 – 4.11)	(3.39 – 3.53)	(2.28 – 2.34)	(1.70 – 1.75)	(1.40 – 1.46)	(1.31 – 1.55)	(2.43 – 2.47)

AMI, Acute Myocardial Infarction.

* Incidence rates are expressed as age-gender group specific event rates per 100 000 person-years of the age-gender group.

¶ Rate differences are differences in incidence rates between men and women, expressed per 100 000 person-years of the age-groups.

**†** Relative Risks are incidence rate ratios with 95 % confidence interval (CI).

‡ Age standardized rates are incidence rates standardized to the European standard population.

**Table 2:** Incidence rates of AMI per 100 000 person-years in the Netherlands between 1997 and 2007, stratified by socioeconomic quintile, age and gender *

	**Age-group (years)**								**Age standardized rate**‡
	**<35**	**35-44**	**45-54**	**55-64**	**65-74**	**75-84**	**85-94**	**≥95**	
**Male**									
Q1 - most affluent	3	58	205	393	704	1146	1606	2057	181
Q2	3	66	235	437	782	1231	1674	2046	200
Q3	4	76	256	474	829	1274	1757	1858	214
Q4	4	84	268	510	883	1317	1752	1852	226
Q5 - most deprived	6	94	308	563	921	1402	1788	1943	246
Total	4	76	252	469	819	1272	1714	1952	212
**Female**									
Q1 - most affluent	1	13	43	94	265	636	1150	1422	68
Q2	1	20	53	119	317	700	1170	1363	79
Q3	1	21	65	134	361	753	1215	1310	88
Q4	2	26	68	157	389	767	1210	1336	94
Q5 - most deprived	2	30	93	193	448	822	1243	1400	108
Total	1	22	63	136	354	737	1199	1368	87

AMI, Acute Myocardial Infarction.

* Incidence rates are expressed as socioeconomic quintile-specific event rates per 100 000 person-years of strata of socioeconomic quintiles within age-gender groups.

‡ Age standardized rates are standardized to the European standard population.

### Socioeconomic inequalities in relative and absolute terms

**Figure 2:**
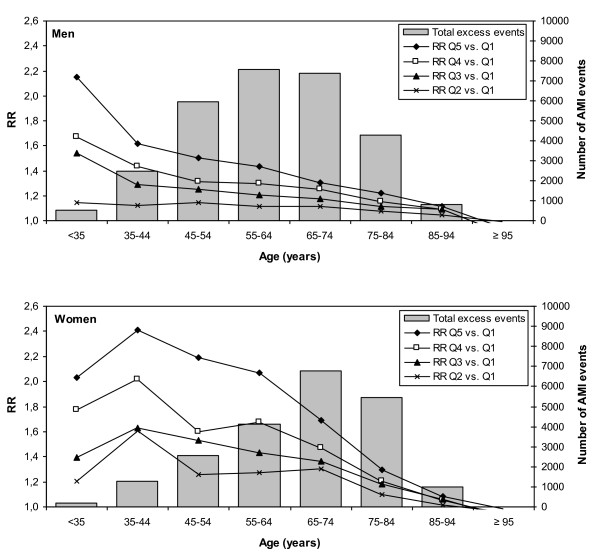
**Socioeconomic inequalities in AMI incidence across age-gender groups in relative and absolute terms.** Relative risks (RRs) for acute myocardial infarction (AMI) per age-gender group by socioeconomic quintile (*lines*) and distribution of excess number of AMI events attributable to socioeconomic inequalities *(shaded bars*) in the Netherlands between 1997 and 2007. RRs are obtained from Poisson regression and compare incidence rates with socioeconomic quintile 1 as reference category. Total excess events are the total number of AMI events in the age-gender groups in the population that would have been eliminated if all had the same risk for AMI as those in socioeconomic quintile Q1.

Table 3 and Figure 2 present RRs for AMI by socioeconomic quintile, stratified by age and gender. There was a clear graded relationship of risk of AMI by socioeconomic quintile. In general, the socioeconomic gradient was steeper in women than men (*P* < 0.001), with an overall RR of 1.34 (95 % CI: 1.32 – 1.36) in men and 1.44 (95 % CI: 1.42 – 1.47) in women, when comparing the most deprived quintile with the most affluent quintile. The association between socioeconomic quintile and AMI incidence decreased with increasing age (Table 3). RRs by socioeconomic quintile appeared highest in women under 65 years and in men under 35 years.

**Table 3:** Relative risks (RRs) and 95 % confidence interval (CI) by socioeconomic quintile for AMI in the Netherlands between 1997 and 2007, stratified by age and gender

	**Age-group (years)**								
	**<35**^A^	**35-44**^A^	**45-54**^A^	**55-64**^A^	**65-74**^A^	**75-84**^A^	**85-94**^A^	**≥95**^A^	**Total**^**B**^
**Male**									
Q1 – most affluent	1	1	1	1	1	1	1	1	1
Q2	1.14 (0.93 – 1.40)	1.12 (1.04 – 1.21)	1.15 (1.11 – 1.19)	1.11 (1.08 – 1.15)	1.11 (1.08 – 1.14)	1.07 (1.04 – 1.11)	1.04 (0.99 – 1.10)	0.99 (0.79 – 1.25)	1.10 (1.08 – 1.12)
Q3	1.54 (1.27 – 1.88)	1.29 (1.20 – 1.39)	1.26 (1.21 – 1.30)	1.21 (1.17 – 1.24)	1.18 (1.14 – 1.21)	1.11 (1.08 – 1.15)	1.09 (1.04 – 1.15)	0.90 (0.72 – 1.13)	1.18 (1.16 – 1.19)
Q4	1.67 (1.40 – 1.94)	1.43 (1.34 – 1.54)	1.32 (1.28 – 1.37)	1.30 (1.26 – 1.34)	1.25 (1.22 – 1.29)	1.15 (1.11 – 1.18)	1.09 (1.03 – 1.15)	0.90 (0.72 – 1.12)	1.24 (1.22 – 1.26)
Q5 – most deprived	2.15 (1.79 – 2.59)	1.62 (1.51 – 1.73)	1.50 (1.45 – 1.55)	1.43 (1.39 – 1.48)	1.31 (1.27 – 1.35)	1.22 (1.19 – 1.26)	1.11 (1.05 – 1.18)	0.94 (0.75 – 1.18)	1.34 (1.32 – 1.36)
**Female**									
Q1 – most affluent	1	1	1	1	1	1	1	1	1
Q2	1.20 (0.86 – 1.69)	1.61 (1.39 – 1.86)	1.26 (1.16 – 1.37)	1.27 (1.20 – 1.36)	1.20 (1.15 – 1.25)	1.10 (1.06 – 1.14)	1.02 (0.97 – 1.06)	0.96 (0.84 – 1.10)	1.15 (1.13 – 1.17)
Q3	1.40 (1.00 – 1.94)	1.63 (1.40 – 1.89)	1.53 (1.41 – 1.66)	1.43 (1.34 – 1.52)	1.36 (1.31 – 1.42)	1.19 (1.15 – 1.23)	1.06 (1.01 – 1.10)	0.92 (0.81 – 1.05)	1.24 (1.21 – 1.26)
Q4	1.77 (1.29 – 2.44)	2.02 (1.75 – 2.33)	1.60 (1.47 – 1.74)	1.68 (1.58 – 1.78)	1.47 (1.41 – 1.53)	1.21 (1.17 – 1.25)	1.05 (1.01 – 1.10)	0.94 (0.83 – 1.07)	1.30 (1.27 – 1.32)
Q5 – most deprived	2.03 (1.49 – 2.76)	2.41 (2.09 – 2.77)	2.19 (2.02 – 2.37)	2.07 (1.95 – 2.19)	1.69 (1.62 – 1.76)	1.29 (1.25 – 1.34)	1.08 (1.02 – 1.13)	0.98 (0.87 – 1.12)	1.44 (1.42 – 1.47)
**Male vs. Female**									
*P*-value ^C^	0.96	<0.001*	<0.001*	<0.001*	<0.001*	0.013*	0.34	0.58	<0.001*

AMI, Acute Myocardial Infarction. *P-value < 0.05.

^A^ Poisson regression model A: socioeconomic quintile, stratified by age and gender.

^B^ Poisson regression model B: socioeconomic quintile adjusted for age, stratified by gender.

^C^ Poisson regression model C: socioeconomic quintile, gender, socioeconomic quintile*gender, stratified by age. *P*-values are obtained from the interaction term socioeconomic quintile*gender.

The total proportion of AMIs that was attributable to socioeconomic inequalities in the Dutch population of 1997 to 2007 was 14 % in men and 18 % in women (PAR; Table 4), corresponding to a total of 50,362 events. Similarly to the RR by socioeconomic quintile the PAR decreased with age. The largest PAR was found in women aged 35–44 with 42 % of the AMI events attributable to socioeconomic inequalities. However, the largest number of excess AMI events attributable to socioeconomic inequalities, measured in absolute numbers, was observed in men aged 45–74 years and in women aged 65–84 years.

**Table 4:** Magnitude of socioeconomic inequalities in AMI incidence in the Netherlands between 1997 and 2007 stratified by age and gender

	**Age-group (years)**								
	**<35**	**35-44**	**45-54**	**55-64**	**65-74**	**75-84**	**85-94**	**≥95**	**Total**
**Male**									
Number of events	1534	10 783	32 120	46 694	52 782	43 502	13 092	714	201 221
PAR (%)**†**	33	23	19	16	14	10	6	N.A.	14
Total excess events**†**	512	2469	5961	7551	7401	4293	825	N.A.	29 011
PP (%)‡	15	10	8	7	5	4	2	N.A.	6
Excess events (ref. previous quintile)‡	237	1043	2550	3227	2744	1690	263	N.A.	11 755
**Female**									
Number of events	561	3032	7863	13 338	25 833	39 597	23 911	2208	116 342
PAR (%)**†**	33	42	32	31	26	14	4	N.A.	18
Total excess events**†**	183	1280	2547	4140	6777	5441	982	N.A.	21 351
PP (%)‡	14	17	15	14	10	5	1	N.A.	7
Excess events (ref. previous quintile)‡	78	508	1195	1849	2571	1881	349	N.A.	8432

AMI, Acute Myocardial Infarction. N.A., not assessed, no significant association between neighbourhood SES and AMI in age-groups **≥**95 years.

**†** PAR: Proportion of AMI incidence attributable to socioeconomic inequalities. Socioeconomic quintile Q1 is the reference category.

Total number of events attributable to total socioeconomic inequalities (= PAR * number of events per age-gender group).

‡ PP: Preventable proportion of AMI incidence attributable to socioeconomic inequalities.

Previous socioeconomic quintile is the reference category.

Number of events attributable to socioeconomic inequalities (= PP * number of events per age-gender group).

We also examined a more realistically attainable preventable proportion (PP) of AMIs that would have been eliminated if all persons from Q2, Q3, Q4 and Q5 would have shifted to the risk for AMI of the next more affluent socioeconomic quintile. Calculated in this manner, the PP was 6 % in men and 7 % in women. This potential reduction in AMI events by reducing socioeconomic inequalities corresponded to a total of 20,187 events between 1997 and 2007, meaning 1,835 preventable AMIs per year. The relative and absolute perspective to socioeconomic inequalities are presented in Figure 2, with an age- and gender-specific comparison of relative risks by socioeconomic quintile with the absolute number of total excess events attributable to socioeconomic inequalities in the Netherlands.

## Discussion

This study adds important new information about neighbourhood socioeconomic inequalities in AMI incidence across a wide range of age- and gender-groups. The combination of relative and absolute perspectives quantifying these age- and gender-variations in socioeconomic differences provides unique information. A considerable proportion of AMI incidence was attributable to socioeconomic inequalities in the Dutch population. The results demonstrated that the increased relative risk for AMI by socioeconomic disadvantage was most apparent in women, as well as in younger persons. In contrast, the largest number of excess AMI events attributable to socioeconomic inequalities was found in middle and early old age.

Our results on incidence rates and the socioeconomic gradient are consistent with other studies of AMI incidence based on neighbourhood-level socioeconomic status [4-6,11,22]. Socioeconomic relative risks were modest, as expected when considering the Netherlands as a relatively small and homogeneous country. The relation between age, gender and the effect of neighbourhood SES on AMI incidence in the Netherlands corresponded to that found in Scottish, Swedish, French and Italian studies [6,11-13], with a steeper socioeconomic gradient in women compared to men, and a decrease in the socioeconomic gradient with increasing age. We can think of several explanations for this age pattern in relative socioeconomic inequalities. Firstly, premature CHD disproportionately affects the most deprived groups. Simultaneously with the increase in the number of AMI events with increasing age, at middle age in men and at early old age in women, the socioeconomic relative risk of AMI started to decrease. High socioeconomic relative risks could be related and limited to premature AMI events. Secondly, socioeconomic inequalities in cardiovascular risk factors are observed to be larger among younger than among older persons, especially regarding smoking [23]. Thirdly, a healthy survivor effect may partly explain the observed decrease in the socioeconomic gradient with age. Selective mortality could narrow socioeconomic inequalities with age since disadvantaged people die younger leaving relatively robust survivors [24,25]. Fourthly, in very old age a substantial part of the population is institutionalized, for whom the neighbourhood of residence might not accurately represent SES.

The apparent “contradiction” between relative and absolute perspectives on socioeconomic inequalities in AMI incidence can be explained by considering the factors that determine the absolute number of excess events attributable to socioeconomic inequalities. Although the socioeconomic gradient in AMI incidence is larger in women and at younger ages, the socioeconomic effect is diluted by the increasing absolute incidence rates with increasing age and male gender. The age-gender structure of the population is the third contributing factor to the absolute number of excess events. We have provided age-gender pyramids to place our findings in the perspective of the demography of the Dutch population. The combination of the three factors resulted in the largest absolute number of excess AMI events attributable to socioeconomic inequalities being found in middle-aged men and middle-aged and elderly women.

This is the first Dutch study to estimate the proportion of AMI incidence attributable to socioeconomic inequalities by using population attributable risk methods [22,26,27]. Hallqvist et al. [27] compared relative and absolute differences in AMI risk according to socioeconomic status in Swedish men and women, based on individual-level SES derived from self-reported occupation. In their study, which concerned a comparison between manual workers and low-level employees with high- and middle-level employees in the age range of 45–64 years, they found a population attributable risk proportion of 17 % in men and 30 % in women over the years 1992–94. The present study found similar results with proportions of 17 % in 45–64 year old men and 32 % in 45–64 year old women attributable to socioeconomic inequalities between 1997 and 2007 in the Netherlands. A study of Ramsay et al. [22] estimated population attributable risks in a population of British men aged 60–79 years old between 1998 and 2000. The population attributable risk for AMI incidence of manual versus non-manual social classes was estimated at 12 %. Men aged 65–74 in the present study showed a population attributable risk of 14 % based on neighbourhood socioeconomic status. However, population attributable risks are difficult to compare across studies. The population attributable risk depends on both the socioeconomic relative risk and on the prevalence of the exposure, in this case the distribution over socioeconomic category. In addition, studies vary widely in their definition of SES. Both individual-level measures (e.g. income, education and occupation) and neighbourhood-level aggregated data or deprivation indices are frequently used to study socioeconomic health differences.

Mean equivalent household income at the neighbourhood-level was used as SES indicator in our study. Income levels have shown to be a good indicator and determinant of SES [28], even in more egalitarian countries [29]. It has been claimed that neighbourhood income has an impact above and beyond the effect that personal income itself exerts on individual health [8,30,31]. The neighbourhood socioeconomic context is thought to contribute to the disadvantage of individuals through material, psychological, physical and social mechanisms [4,32,33]. In addition, the effect of neighbourhood SES can be in part either due to or mediated through conventional risk factors [34]. For example, prevalence of smoking, obesity and physical inactivity were found to be higher among more deprived populations in Sweden, independent of individual-level socioeconomic status [3]. Approximately 50 % of the relative and absolute socioeconomic difference in CHD risk can probably be explained by the four behavioural and biological risk factors - hypertension, smoking, high cholesterol and diabetes [26,35,36].

This nationwide study has several strengths but also some limitations. Strengths are its large size, population-based nature and the wide range of age- and gender-groups studied. A limitation is that the Dutch hospital discharge register was digitally available for record linkage only from registration year 1995 onwards. Most recurrent events occur within one year after the first AMI events [37], although some AMI events, particularly in the beginning of the study period could have been misclassified as being incident events. Because the socioeconomic gradient in AMI risk has been reported to be of similar magnitude in recurrent and incident AMI events [38], we did not consider this limitation a problem. A second limitation to address is that neighbourhood SES was assessed only at a single point in time based on the first place of residence in the study period. Neighbourhood of residence may have changed during follow-up. Moving out of areas might have diluted the effect of neighbourhood to a small extent, although most residential mobility in the Netherlands is to neighbourhoods with comparable neighbourhood socioeconomic status [39]. Besides the effect neighbourhood itself exerts on health, neighbourhood SES might also serve as proxy for individual-level SES. Unfortunately, our nationwide study did not allow us to disentangle the neighbourhood, individual and behavioural and biological effects captured by neighbourhood-level SES.

Population attributable risks can inform policy makers in planning public health interventions [40]. Nonetheless, some caution should be taken in the interpretation of population attributable risks associated with socioeconomic inequalities. The starting point of population attributable risk calculations is the assumption that there is a causal relationship between exposure and disease. Since conventional risk factors may mediate rather than confound part of the effect of neighbourhood SES on CHD [10,41], we considered the method appropriate to estimate the number of potentially preventable AMI events attributable to socioeconomic inequalities. With our estimates of the relative risks, population attributable risks and absolute numbers of excess events due to socioeconomic inequalities we have provided information which can be used in prevention at the individual level, and ultimately, to improve population health. It is not realistic to expect that the total estimated population attributable risk proportion, that was attributable in the past, could be avoided entirely in the future. This would essentially mean to eliminate all inequality. Therefore we have also estimated the potential impact of a population shift in the risk for AMI associated with socioeconomic inequalities, adopting a population approach. Public health policies aimed at reducing socioeconomic inequalities in AMI incidence should take note of the considerable benefit of shifting the population distribution, even in seemingly egalitarian countries.

The implications for CHD prevention across the life course are clear. AMI incidence is powerfully influenced by past as well as present socioeconomic status. Effective interventions early in the life course might ameliorate risk factors of CHD before irreversible vascular damage has occurred. Nevertheless, middle-aged and older persons currently suffer from the largest burden of disease attributable to socioeconomic inequalities. Prevention programs with rapid benefits, such as smoking cessation and dietary change, should therefore not be overlooked.

## Conclusions

Neighbourhood socioeconomic inequalities were observed in AMI incidence in the Netherlands, but the magnitude across age-gender groups depended on whether inequality was expressed in relative or absolute terms. Relative socioeconomic inequalities were high in young persons and women, where the absolute burden of AMI was low. Absolute socioeconomic inequalities in AMI were highest in the age-gender groups of middle-aged men and elderly women, where the number of cases was largest.

## Abbreviations

AMI: Acute myocardial infarction; CHD: Coronary heart disease; CI: Confidence interval; HDR: Hospital discharge register; PAR: Population attributable risk proportion; PP: Preventable proportion; RR: Relative risk; SES: Socioeconomic status.

## Competing interests

The authors declare that they have no competing interests.

## Authors’ contributions

IV is the corresponding author and guarantor of this paper. CK, IV and MLB formulated the research question and initiated the study. CK and AAMvO performed the registry linkage. CK analysed the data and wrote the paper. AAMvO, MLB, PME, WMMV, LvR, IvD, SC and IV made substantial contributions, helped with interpretation of the data and commented on the paper as well as revisions to drafts of the paper. All authors read and approved the final manuscript.

## Pre-publication history

The pre-publication history for this paper can be accessed here:

http://www.biomedcentral.com/1471-2458/12/617/prepub

## Supplementary Material

Additional file 1 Explanation and example calculation of Population Attributable Risks (PAR) and Preventable Proportion (PP). Click here for file
