# Supplementary material for: Neighbourhood socioeconomic inequalities in incidence of acute myocardial infarction: a cohort study quantifying age- and gender-specific differences in relative and absolute terms
Source: BMC Public Health. 2012 Aug 7;12:617. doi: 10.1186/1471-2458-12-617 (PMC3490806; doi:10.1186/1471-2458-12-617)
Supplement: Additional file 1 — Explanation and example calculation of Population Attributable Risks (PAR) and Preventable Proportion (PP). [file 1471-2458-12-617-S1.pdf]

Additional File 1: Explanation and example calculation of Population Attributable Risk (PAR) and Preventable Proportion (PP)

Relates to “Neighborhood socioeconomic inequalities in incidence of acute myocardial infarction: a cohort study quantifying age- and gender-specific differences in relative and absolute terms” by Koopman et al.

Schematic presentation using artificial data:

Incidence and proportions of PAR, PP, explained by relative risks (RR) on Y-axis and proportion of quintiles on X-axis.

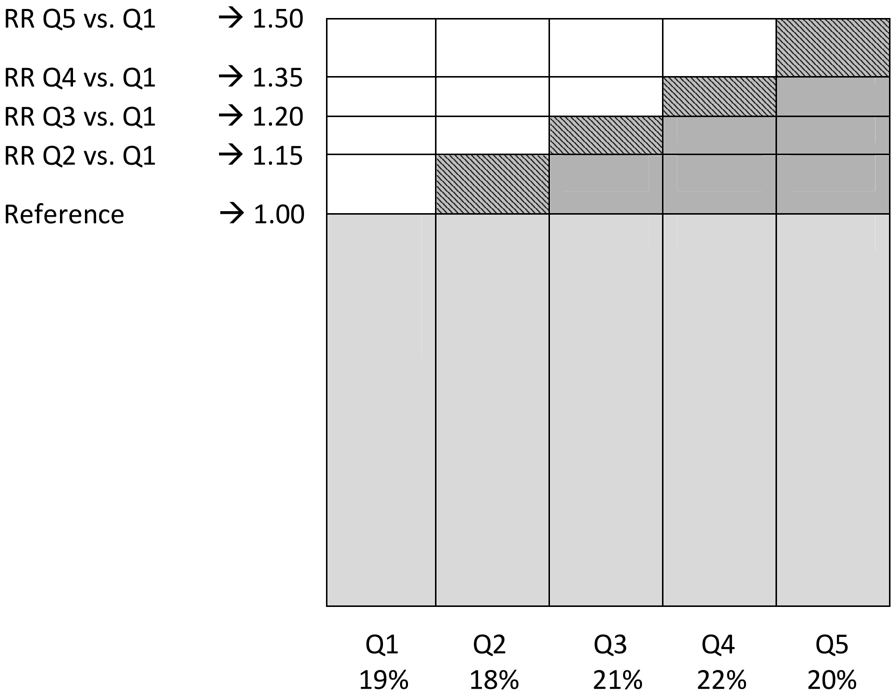

Legend

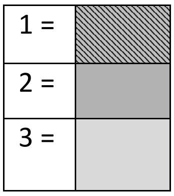

Preventable proportion (PP) = 1 / (1+2+3)

Population Attributable Risk proportion (PAR) = (1+2) / (1+2+3)

Total incidence = 1+2+3

PAR example calculation:

$$\text{PAR} = \frac{\sum_{i=0}^{k} p_i (RR_i - 1)}{1 + \sum_{i=0}^{k} p_i (RR_i - 1)}$$

$$\text{PAR} = \frac{0.18*(1.15-1) + 0.21*(1.20-1) + 0.22*(1.35-1) + 0.20*(1.50-1)}{1+ (0.18*(1.15-1) + 0.21*(1.20-1) + 0.22*(1.35-1) + 0.20*(1.50-1))} = \frac{0.246}{1+ 0.246} = 0.197 \rightarrow 19.7\%$$

PP example calculation:

$$\text{PP} = \frac{\sum_{i=1}^{k} p_i (RR_i - RR_{i-1})}{1 + \sum_{i=0}^{k} p_i (RR_i - 1)}$$

$$\text{PP} = \frac{0.18*(1.15-1) + 0.21*(1.20-1.15) + 0.22*(1.35-1.20) + 0.20*(1.50-1.35)}{1+ (0.18*(1.15-1) + 0.21*(1.20-1) + 0.22*(1.35-1) + 0.20*(1.50-1))} = \frac{0.1005}{1+ 0.246} = 0.081 \rightarrow 8.1\%$$
